# First Partner Choice in a Native Minority: The Role of Own and Parental Ethnolinguistic Affiliation

**DOI:** 10.1007/s10680-023-09683-2

**Published:** 2024-01-16

**Authors:** Caroline Uggla, Jan Saarela

**Affiliations:** 1 Demography Unit, Department of Sociology, Stockholm Univeristy, Stockholm, Sweden; 2 Åbo Akademi, Vasa, Finland

**Keywords:** Partner choice, Endogamy, Minority-majority union, Ethnicity, Cohabitation

## Abstract

Despite increasing diversity within many societies, ethnically endogamous unions remain common. In contexts where one ethnic minority has lived alongside the majority for centuries, understanding who partners with whom is central to understanding how ethnic boundaries are maintained or dissolved. This study examines the role of own and parental ethnolinguistic affiliation for the first partner choice in Finland. We provide a unique test of the relevance of ethnic endogamy across two generations, in a context where both groups are native, but one (Finnish speakers) overwhelmingly outnumbers the other (Swedish speakers). Using register data on the total population, we examine how a person’s ethnolinguistic affiliation and background affect the choice of the first cohabiting partner in terms of the partner’s ethnolinguistic affiliation and background. We apply discrete-time competing risk models for men and women born 1970–1983. Results indicate that Swedish-registered individuals with two Swedish-registered parents are, by far, the most likely to partner with another Swedish-registered person with endogamous background. Partnering with a Swedish-registered person with exogamous background is most likely among individuals who themselves come from mixed unions. Patterns are remarkably consistent across gender, and adjustments for education and residential area only marginally alter the results.

## Introduction

When individuals from minority groups find partners in the majority population, social boundaries are blurred, and minority group belonging may be weakened over time, or even disappear. Understanding how individuals from minority groups navigate the partner market is, therefore, essential to comprehend the process of intergenerational transmission of ethnic identities. This question is highly relevant given that heterogeneity has increased within many populations through recent waves of immigration to North American and European countries, the so-called diversity explosion (Frey, 2014). As a consequence, more individuals are growing up with parents with different ethnicities (Andersson et al., 2015; Kulu & González-Ferrer, 2014). In many places, increased modernization and urbanization have occurred in conjunction with a gradual break-down of social boundaries between groups, and those most affected by modernization are expected to be most likely to intermarry (O’Leary, 2001). Many people make partner decisions on emotional rather than instrumental reasons (Shorter, 1975) and can independently decide with whom to enter a cohabiting union. These processes are thus associated with lesser influence of third parties, such as parents or social or religious institutions. In contemporary societies, people increasingly meet in new arenas, such as educational establishments, where they might assort on achieved traits, such as education, rather than on ascribed traits, such as ethnicity (Blossfeld, 2009).

Yet, individuals assorting on ethnicity is a robust finding across the social sciences (Hwang et al., 1997; Kalmijn, 1994; Kalmijn & van Tubergen, 2006). This pattern seems to persist even in heterogeneous populations, and where an ethnic minority is small and the odds are stacked against endogamy. However, much of the literature on determinants of partner choice among majority-minority groups is based on native-immigrant unions, where intermarriage is considered the final step in the integration process (Litcher & Qian, 2019). Little of what is known about mixed unions is based on partnership between two native or indigenous groups, especially in a European context (Obućina, 2016; Saarela & Finnäs, 2014). Understanding union formation in the context of native minority-majority groups is crucial, as it may shed light on how groups who have lived side-by-side for centuries maintain social boundaries and how ethnicity is passed on. With increasing prevalence of mixed unions in many contexts, an increasing amount of individuals have an exogamous background, that is, have parents who are discordant on a given trait. Despite this development, most studies base partner characteristics on a single measure, such as ego’s ethnicity, and risk discounting the impact of mixed parental ancestry, and/or how affiliation of the children interacts with the parents’ affiliation in shaping partner choice for the next generation.

Compared to native-immigrant intermarriages, much less is known about partner choice in contexts where the minority is not an immigrant group. This is important for several reasons. First, when exogamy is defined as between two distinct ancestral groups, this provides insights into how ethnicity is passed on across generations, and how individuals navigate group belonging (partner preference) in tandem with timing of life events such as partnership formation. In contrast to native-immigrant partnerships, exogamous unions between two native groups do not suffer from migration-event biases and therefore avoid issues of how to interpret marriage migration. Second, when both ancestral groups have social contexts and the same established relations that many immigrants lack when they settle in a destination country, the comparison between the groups becomes more equal. Individuals in both native minority and majority groups have grown up with knowledge and presence of the other group, and may share political and regional aspects, as a result from being part of the same nation state. Third, when the minority group is neither economically, nor socially disadvantaged, any potential bias from selection on social status or resources, and exogamy through status exchange, is removed. Status exchange is generally taken to mean that partners trade desirable characteristics in one domain (e.g. ethnicity, age or being never-married) for another (e.g. higher social status or higher household income). Some of the arguments outlined above for why it is crucial to study separate native-born groups could be applied to studies of descendants of immigrants, for whom there is a growing literature (Kulu & González-Ferrer, 2014). Yet, here we argue that there is an essential distinction between a minority group with parents who are native-born and descendants of immigrants who have an ancestral country and ethnicity based elsewhere.

A longstanding issue for sociologists seeking to study partnerships is how to best capture the unions an individual might have over his or her life course. Asking respondents later in life is related to recall bias and variations in definitions of partnerships. However, focusing solely on marital unions overlooks cohabitations, which are increasingly important as long and stable unions in many contexts. Survey data often fail to capture partnerships with accuracy, while register-based studies which also comprise data on ethnicity are rare. To our knowledge, there are no previous studies on how parental ethnic affiliation is associated with partner choice in a reliable complete population dataset that captures all cohabitation unions and marriages alike.

In this paper we seek to address this gap in the literature by drawing on unique world-class data on union formation from Finland. The focus of the paper is to examine with whom individuals enter their first cohabiting union in Finland, where the Swedish-speaking minority (5% of the population) resides next to the Finnish-speaking majority (87%) and has done so for centuries. Finland is a unique context with two distinct native ethnolinguistic groups with equal constitutional rights, basically no discrimination based on ethnolinguistic affiliation, and intermarriage across the two groups is common (see Saarela, 2021). That the social barriers between Swedish and Finnish speakers are low, together with the egalitarian and homogenous context, is important when it comes to transferring any dynamics to relationships between other social groups, and their ability to break group boundaries. There are examples in this realm, for instance on ethnic intermarriage between ancestral natives in former Yugoslavia (Smits, 2010), and religious intermarriage between and Catholics and Protestants in Northern Ireland (O’Leary & Finnäs, 2002), but there is a notable lack of examples where exogamous unions suffer little stigma, discrimination or other social sanctions.

Here we are primarily interested in the partner choice of Swedish-registered individuals (with uniform Swedish background or mixed Swedish-Finnish background), because it is the behaviour of these individuals that will determine how the Swedish-speaking identity is passed on, and the future position of the Swedish language in Finland. That a significant proportion of Swedish speakers enter exogamous unions is in itself a reason for why greater understanding of their partner choice is desirable. Our contribution will display the extent to which endogamy is maintained for the Swedish-speaking minority with a fully Swedish-speaking background, and also the patterns in partner choice for Swedish speakers with mixed background. We adjust for contextual factors, such as the share of Swedish speakers in the local area, and educational level of the index persons and their parents. Most notably, we examine partner choice not only by an individual’s own ethnolinguistic affiliation, but also by both parents’ ethnolinguistic affiliation.

## Background

Endogamy or homogamy, that two partners share ascribed or achieved characteristics, is common. Assortative mating based on age, education, ethnicity and religion is prevalent in many contexts (Blossfeld, 2009; Carol, 2016; O’Leary & Finnäs, 2002; Qian & Lichter, 2018; Wiik & Holland, 2018). Ethnicity and race are two dimensions that show considerable homophily in friendships, as well as marriages (Mcpherson et al., 2001). Despite great diversity within many contemporary societies, especially with the current levels of international immigration, a considerable proportion of unions formed are still endogamous in one way or another (Hannemann et al., 2018). Such matching might occur when individuals prefer a partner similar to themselves. A large body of literature has documented that unions where partners share characteristics or have greater “value similarity” are both more common and more stable (Dribe & Lundh, 2012; Kalmijn et al., 2005; Milewski & Kulu, 2014; van Ham & Tammaru, 2011). Ethnic endogamy is of high sociological relevance as it can provide an indication of how close different groups are to one another, and how these relations and attitudes may change over time. When the minority is an immigrant group and the majority the mainstream population, the research question often invokes assimilation, and views intermarriage as the final step of the integration process.

So why does endogamy arise? It is generally argued that partner choice is governed by preferences, opportunities and third party norms (Kalmijn, 1998). An individual might hold certain preferences for a putative partner, but whether these are realized is contingent on the supply of partners who meets one’s criteria (Blau & Schwartz, 1984). Opportunities can be operationalized as the absolute number of potential partners within a given group, the relative size of ethnic groups, as well as the adult sex ratio and the level of segregation between social or ethnic groups (Kulu & González-Ferrer, 2014). Studies that have sought to examine opportunities for minority groups have often focused on migrant groups in the US or Europe, and examined their relative group size and likelihood of exogamy, in terms of partnership with the mainstream population or another immigrant origin group. For example, among minority immigrant groups in the Netherlands, origin group size is negatively correlated with ethnic exogamy (van Tubergen & Maas, 2007). That is, a larger origin group size means a greater likelihood of marrying within one’s own group. Although the world is becoming increasingly interconnected and the opportunities to meet partners may have increased (the pool has expanded), many people still find a partner who lives nearby (Haandrikman et al., 2011), or attends the same institutions, such as higher education (Blossfeld, 2009), and therefore often are like themselves (Schwartz & Mare, 2005). Evidence even suggests that with the advent of online dating, couples have become more endogamous, because finding others of the same ethnicity is facilitated by the online search tools (Thomas, 2020).

In addition to individual preferences and opportunities, norms regarding whom to partner with matter too. Third party influence from parents has been a focal decision-maker in marriages across the globe historically, when arranged marriages and material transactions between lineages were common (Fox, 1967). In many non-western cultures, parents still have a large say in marital arrangements, although this influence has lessened over time with modernization and marriage for emotional rather than instrumental reasons (Shorter, 1975). Nevertheless, even in contemporary Western societies, crossing social boundaries in marriage and unions is generally associated with some degree of normative disapproval (Kalmijn et al., 2005). A lack of support and encouragement from family and friends within one’s group may explain higher rates of divorce among mixed unions, and why such unions are less likely to be favoured in the first place. Religious institutions and social ties in small communities have also been important in preserving and promoting norms of endogamy. Many young adults leave the nest and enter a more independent life phase where parents are not able to interfere, and can choose their own social circles. For instance, young adults in the USA who move further away from their parents are more likely to enter racial exogamous unions than those who remain geographically closer to their parents (Rosenfeld & Kim, 2005).

### Matching and Other Mechanisms

Sociologists and other scholars have attempted to tease apart the different mechanisms that can give rise to endogamous (or homogamous) unions. The matching hypothesis postulates that individuals seek others who are like themselves (DiMaggio & Mohr, 1985; Kalmijn, 1994). Studies that compare intermarriage between different ethnic or immigrant origin groups repeatedly find that groups that are more closely related in terms of values are more likely to intermarry (Dribe & Lundh, 2011; van Ham & Tammaru, 2011). But sharing the same ethnolinguistic background may be seen as a particularly poignant trait in a prospective partner, as it not only signifies group belonging but also eases communication.

Such examples of assortative mating may also arise due to competition. The competition hypothesis posits that individuals seek the highest possible amount of a given trait (Mare, 1991). If most individuals favour a highly educated partner, educational homogamy can result from the fact that those with the highest education themselves are more likely to be favoured by other highly educated individuals. When examining these explanations in a Western or European context, support has been found for competition on economic traits, but matching on cultural traits (Kalmijn, 1994; Schwartz, 2013). In contrast, exogamous unions, where individuals differ on ascribed traits, may arise because partners exchange traits, meaning that a more desirable characteristic in one domain is traded for a less desirable characteristic in another (Merton, 1941), such as ethnicity for high education or income. Status exchange theory originated from studies on Black-White intermarriage in the USA, but empirical support for it is more ambiguous for other ethnic groups and contexts (Jacobs & Labov, 2002; Kalmijn, 2010; Kalmijn & van Tubergen, 2006). Inherent to the idea of status exchange is a clear hierarchy between ethnic groups. This is less relevant when, as in this study, categories are nominal, i.e. there are two socially equal groups who both might seek to find a culturally similar partner who speaks the same language.

Determining the mechanisms that underpin partner choice has proven to be a tricky feat. Our main objective here is not to conclusively determine the mechanisms between partner choice based on ethnolinguistic identity. Rather, due to our unique data, we are able to ascertain how and why partner choices differ when taking into account ethnicity across multiple generations. By controlling for education of ego and his/her parents and the language mostly spoken in the residential area, we are able to provide evidence of whom is most likely to break the language barrier, and who is most likely to maintain it, within both the majority and the minority group.

## The Study Context

There are few contexts where partner choice in a constrained partner market can be studied through population-wide data with a high degree of resolution. Finland provides a unique exception in this respect. The country has two ancestral native ethnolinguistic groups, Finnish speakers (87%) and Swedish speakers (5%, or approximately 290 000 individuals). Finnish and Swedish come from different language trees, are highly distinct, and are not intuitively understandable to each other as Swedish, Norwegian and Danish are. While the two groups have the same constitutional rights and are similar on many observable characteristics (Saarela & Finnäs, 2014), the ethnolinguistic division has profound impact on Finnish society, through separate social and cultural institutions, parallel school systems, geographic residential segregation, and even a separate Swedish-speaking army brigade (McRae, 1997). This ethnolinguistic division stems from centuries of shared history, as Finland was a part of the Swedish realm until 1809, when it fell under Russian rule. When Finland became independent from the Russian empire in 1917, it was as a bilingual republic in which the two groups were guaranteed equal rights. The two ethnolinguistic groups in Finland function like separate ethnicities in how they are traditionally defined (cf. Gordon, 1964). They are also divided by the practicalities of two distinct languages that do not share recent linguistic roots.

Since the 1950s the Swedish-speaking population has been facing large demographic changes. Swedish speakers have decreased in relative as well as absolute terms, Finnish speakers have moved into regions that were previously primarily Swedish-speaking, and the proportion of individuals who find their partner across the ethnolinguistic border has doubled (Finnäs, 2012). In the 1950s, approximately 20% of the Swedish-speaking population married a Finnish-speaking spouse (Finnäs, 1986). This figure rose gradually until the 1980s when it levelled off, and today about 40% of the unions of Swedish speakers are to a Finnish speaker (Saarela, 2021). Given that this is a significant proportion of all Swedish-registered people, it is important to understand the causes and consequences of these unions.

### Ethnolinguistic Registration and Identity

All Finnish-born persons are registered with a mother tongue. This ethnolinguistic registration is generally done recently after birth. The population registration system enforces a binomial view of the ethnolinguistic boundaries in the sense that multiple affiliations cannot be chosen. Having one Finnish-speaking parent who has chosen or agreed to register the child as a Swedish speaker seems to lead to a “Swedish-speaking identity”. Note that Swedish speakers are not immigrants from Sweden, but rather a long-standing native ethnic group. Thus, while the language is likely spoken, parents’ choice of language is not associated with any binding requirements, as denied access to a preschool or school where the other language is spoken. As such, language registration may be taken as an expression of “symbolic ethnicity” (Gans, 1979).

It is possible to change language in later life, but to do so is very rare. In our sample, only 0.7% have ever switched from Swedish to Finnish or the other way around. Even this low figure is an overestimation of language shifts, because children born late in the year are automatically registered with the mother’s registered language which may be updated in the following calendar year. (The underestimation of Swedish-registered newborns is estimated to be about 5% (Saarela, 2021)). In Saarela et al. (2022) language switching is estimated to about 0.06%. Earlier studies have argued that the mother tongue can be interpreted as close to fixed ethnicity or ethnolinguistic identity (Finnäs, 1986). Malleability of ethnolinguistic identity is therefore low and any language switching can be deemed too low to use for analyses (Finnäs, 1997). On a practical level, most children in mixed families are able to speak both Swedish and Finnish, and may use them both in the home and in society at large. While both languages are mandatory in school, knowledge of the other language is often poor among Finnish speakers, whereas most Swedish speakers are able to communicate in the Finnish language. Apart from residents of the island of Åland, almost all Swedish speakers born after World War II, are proficient in both languages (Obućina & Saarela, 2020). Swedish speakers who reside in mixed regions, such as the Helsinki area, are likely to speak both languages well, whereas bilingualism is less common among those who are registered as Finnish speakers, and particularly so outside the Swedish speakers main settlement area along the southern and western coastline.

Approximately 65% of all children born in Finnish-Swedish unions are currently registered as Swedish speakers. If it is the mother who is Swedish-registered, this proportion is almost 85%, while it is about 55% if it is the father who is Swedish-registered (Saarela, 2021). Both gender and education matter for language registration. In exogamous unions, mothers are more likely to pass on their ethnolinguistic affiliation to children than fathers, while Swedish-speaking men are more likely to partner with Finnish-speaking women, than vice versa (Saarela et al., 2020). Finnäs and O’Leary (2003) have shown that the education of both the Finnish-speaking and the Swedish-speaking parents impact the language registration of their child, but the education of the Swedish-registered parent has greater impact. If the Swedish-speaking parent is highly educated, the couple is more likely to register the child as Swedish-speaking.

An increase in the number of unions across the ethnolinguistic border during the twentieth century has meant that a substantial number of children are raised by parents from both ethnolinguistic groups. However, there are clear differences in the stability of unions between endogamous Finnish-speaking and Swedish-speaking unions. Endogamous Finnish-speaking unions have about twice as high separation risk as endogamous Swedish-speaking unions, and these differences cannot be explained by socioeconomic differences between the groups (Finnäs, 1997). Highest of all was the divorce risk of exogamous unions, with circa 10% higher risk than endogamous Finnish unions. Arguments about high social integration and low mobility of Swedish speakers have been proposed as mechanisms behind the stability of Swedish-endogamous unions (Finnäs, 1997; Saarela & Finnäs, 2018). Out of all compositions, ethnolinguistically exogamous unions are thus the most labile (Finnäs, 1997; Saarela & Finnäs, 2014), which suggests that individuals from both ethnolinguistic groups pay some cost from partnering outside of their own group. Research also suggests that language can be a barrier of interaction for couples (Saarela et al., 2022).

Although the two groups have become closer and more intermixed, Finland is currently at a juncture where there is still a clear divide between the majority Finnish speakers and the minority Swedish speakers. While closer integration across social groups in a society is clearly beneficial to social cohesion, it is not known how the present dynamics will impact the long-term development of the Swedish-speaking minority. How an ethnic minority will fare in relative numbers is determined by demographic processes related to births, deaths and emigration. Birth rates and death rates currently have a negligible impact on Finnish/Swedish population composition, while net emigration rates have a slightly more prominent role (Weber & Saarela, 2019). The single most important factor is instead the extent and patterning of exogamous partnership, and in particular how the ethnolinguistic affiliation in these are passed on to the next generation. However, most research has been based on ego’s (single) measure of ethnolinguistic affiliation, and little is known about how partner choice differs by own and parental ethnolinguistic affiliation (Finnäs, 2015).

## Contribution

In this paper, we make three key contributions. First, we are able to closely examine how individuals with exogamous background maintain, or further dissolve, group boundaries by using data on ethnolinguistic identity across two generations. In the international literature, data on ethnic group belonging across generations have been rarely used (but see Dribe et al., 2018 for a historical example), and individuals with mixed heritage have often been inferred as the product of an assimilation process, without dissecting the majority versus minority perspective in the own partner choice. An exception is an examination from Sweden, demonstrating that individuals with one foreign-born parent are more likely to enter intermarriages than individuals with endogamous Swedish backgrounds (Irastorza & Elwert, 2021). Highly detailed data on ethnolinguistic affiliation of the ego and of both parents, is rare but necessary for the fine-grained groups between which we distinguish. The focal individuals who are Swedish-registered may have two Swedish-registered parents (uniform Swedish background), or have exogamous parents (i.e. have mixed Swedish/Finnish background). The same goes for individuals who are Finnish-registered (F-uniform Finnish background or F-mixed Swedish/Finnish background), leading to four distinct categories of ethnolinguistic affiliation. (Hereafter we refer to these categories as S-uniform, S-mixed, F-uniform, F-mixed, where the capital letter denotes egos affiliation and mixed/uniform the parental language background). Note that parents need not be Finnish-born but have to have resided in Finland at some point for us to know their registered language. Second, we have complete population data on all cohabiting unisons. This allows us to capture all long-term relationships, not just marriages. While our ego index persons are those who are born in Finland, we also include partners who have immigrated, as to include all possible first partner choices of our focal individuals. Third, we control for educational level of both ego and their parents, as well as the ethnolinguistic composition and sex ratio of the area of residence area, so that differential geographic opportunities to find a given partner should not bias the results. If there are differences between educational groups in endogamy/exogamy in Finland, we expect partner choices to shift when we adjust for education across two generations. In contrast, if ethnolinguistic identity is the main bearing factor, we expect patterns in partner choice to remain unchanged even after adjustment for ego and parents education.

## Predictions

We depart from the matching hypothesis and predict that Swedish-registered individuals with two Swedish-registered parents (S uniform) will be most likely to partner with others with the same composition (S uniform), followed by Swedish-registered persons with mixed S/F backgrounds. Then follow Finnish-registered persons with mixed backgrounds, and lastly Finnish-registered persons with uniform Finnish background (F-uniform).

For Swedish-registered individuals with uniform Swedish background, we thus predict that partners will be in the order:

$${\text{S}} - {\text{uniform }} > {\text{ S}} - {\text{mixed }} > {\text{ F}} - {\text{mixed }} > {\text{ F}} - {\text{uniform}}$$

Similarly, we predict that a Finnish-registered ego with uniform Finnish background will be most likely to partner in an inversed pattern compared to the Swedish-registered persons above, i.e.:

$${\text{F}} - {\text{uniform }} > {\text{ F}} - {\text{mixed }} > {\text{ S}} - {\text{mixed }} > {\text{ S}} - {\text{uniform}}$$

For individuals who have mixed backgrounds, two different sets of predictions can be made: matching based on their own registered language, and matching based on the shared parental background. Given that own registered language is a reliable predictor of group belonging in this context, we predict that Swedish-registered individuals with mixed backgrounds will be most likely to partner with:

$${\text{S}} - {\text{mixed }} > {\text{ S}} - {\text{uniform }} > {\text{ F}} - {\text{mixed }} > {\text{ F}} - {\text{uniform}}$$

Accordingly, for Finnish-registered egos with mixed background, we predict partner choice in the following order:

$${\text{F}} - {\text{mixed }} > {\text{ F}} - {\text{uniform }} > {\text{ S}} - {\text{mixed }} > {\text{ S}} - {\text{uniform}}$$

If instead S-mixed would be more likely than F-uniform individuals, this would imply that partner choice is governed primarily by parental background, than the individual’s own language.

A partner outcome “other” consisting of all other potential ethnolinguistic combinations, is included for completeness, but we make no a priori predictions about where it will fall. Importantly, in addition to the predicted ranking described above, our empirical analyses will reveal any differences in magnitude between the ethnolinguistic categories. Results will therefore disclose if the closeness in partner choice is gradually driven by the degree of ethnolinguistic affiliation, or whether there is binary divide between Swedish- and Finnish-registered persons. Lastly, we will also examine magnitude differences by gender. Previous research has shown that Swedish-registered men are more likely to partner with a female Finnish-speaker than the reverse (Saarela & Finnäs, 2014). However, it is not known how this plays out when data over two generations are considered. Based on previous knowledge, we predict that tendency for exogamous partnerships is higher for Swedish-registered men than for Swedish-registered women.

## Data and Methodology

We use Finnish register data that have unique linkage of ethnolinguistic identity for multiple generations. Each person in the data can be linked to his or her mother and father, as long as the parent had not died before the end of 1970. Through anonymized person numbers we can link individuals to various socioeconomic variables and demographic controls, and importantly to cohabitation by the residential address. The data is accessed through Statistics Finland’s FIONA system, and used with the permission number TK-53–1370-17.

In the analyses, we include all individuals who were born in Finland 1970–1983, and who have information on their own, mother’s and father’s registered mother tongue (Finnish, Swedish, or other). All individuals have information about their unique mother tongue in the population register. We impose the restriction that the individual must be resident in Finland from birth until age 18, when we start the time at risk. The oldest individuals (born in 1970) will begin their time at risk in 1988 and are followed until age 35 in 2005. The youngest cohort (born in 1983) will be 35 in 2018, which is our last year of observation. The partner choice measured is ego’s first cohabiting partner. Cohabitation is wide-spread in Finland, and many such unions subsequently turn into marital unions (Saarela & Finnäs, 2014).

Finland is one of the few countries in the world where cohabiting unions, regardless of whether the couple has children or not, can be identified in the population registers. Cohabitations are based on a definition by Statistics Finland that notes if a person is domiciled with an opposite-sex individual (we can consider heterosexual couples only) who is not a sibling or a parent, in the same dwelling beyond 90 days, and the age difference to the other person does not exceed 20 years. We recognize that non-couples in shared housing (e.g. roommates) may fall into this category, but this number is small and many students continue to be registered with their parents. Cohabitation is also recognized if the couple has a common child. We include all cohabitations, that is, also those that start as marital unions, although for women born in the 1960s-1980s, only 10% of all unions started with marriage (Jalovaara, 2012). The cohabitation measure applied has been established as accurate (Lyngstad & Jalovaara, 2010), and conforms to international standards for the classification and identification of couples in households (Kennedy & Fitch, 2012).

### Ethnolinguistic Affiliation

The measure of ethnolinguistic affiliation refers to the ego’s, the mother’s and the father’s mother tongue, as observed in the population register. Ego’s mother tongue is measured at age 18. Few individuals change their registered mother tongue after this point (Obućina & Saarela, 2020). In our data, only 0.7% have ever switched from Swedish to Finnish or vice versa. For the parents, the language refers to whether a person has ever been Swedish-registered/ever Finnish-registered (cf. Saarela et al., 2020). This typology results in four categories for the egos: S-uniform, S-mixed, F-mixed and F-uniform. All other, and generally uncommon, combinations are excluded as there are almost no individuals who are themselves Finnish-registered but who have two Swedish-registered parents (circa 0.08% in our data). Given how rare these individuals are, it is not possible to interpret this category as self-identification or to use it for a robustness check; they are more likely protest registration or data inconsistencies. The same typology is used for partners’ ethnolinguistic affiliation, however for partners we include a “other” category in order to capture all possible partner choices. Partners’ parents who have missing data on language registration, are coded as “other”. “Other” is comprised predominantly by foreign-born individuals with some other mother tongue than Swedish or Finnish, and has been small until recently due to the low number of foreign-born immigrants before the 1990s.

Note that 4.4% of egos in our study have their first cohabiting union with a partner who was born abroad. Among Swedish-speaking egos, the figure is around 7.6%. However, among couples where both the ego and the partner are Swedish-speakers, only 3.0% of the partners are foreign-born. This figure should not be taken to represent “partner import” (e.g. of Swedish-speakers who are Swedish citizens) for several reasons. First, some foreign-born individuals will move to Finland during childhood; they immigrate for reasons other than partnership formation. Second, another group of foreign-born partners will be children of Finnish nationals who are Swedish-speaking, but have resided some time abroad. In our data, over 86% of foreign-born partners who are Swedish-speaking have at least one Finnish-born parent. Among all foreign-born partners in our study population (regardless of their language registration) about 40% have a Finnish-born parent. Moreover, the descriptive statistics show that it is common to leave Finland in early adulthood, especially among the Swedish-speaking minority (see Table 1). Thus, as context to the herein presented analyses, it is much more common that Swedish-speakers emigrate to Sweden (and may find a partner there eventually), than the other way around.

**Table 1:** Women’s first cohabiting partner by own ethnolinguistic background (*n* = 404 620)

		Partner’s ethnolinguistic background/censoring outcome								
Ego’s ethnolinguistic background		S-uniform (%)	S-mixed (%)	F-mixed (%)	F-uniform (%)	Other (%)	Never cohab. (%)	Emigrated (%)	Died (%)	Total
	S-uniform	45.7	9.9	2.5	15.1	2.6	6.8	17.2	0.2	14 653
	S-mixed	23.5	8.6	3.7	41.4	3.7	8.1	10.9	0.2	7 374
	F-mixed	5.6	4.2	4.1	69.3	3.5	8.1	5.0	0,3	7 634
	F-uniform	0.8	0.9	1.5	82.8	2.6	8.1	3.1	0.3	374 959

S/F-Mixed background refer to those with one parent who is Swedish-speaker and one who is a Finnish-speaker, S/F-uniform refer where both parents are registered with the same language as ego. If no cohabitation has occurred by age 35, the individual is recorded as never cohabitated. Emigrated and died are recorded as such if this event occurs before any cohabitation. Individuals who return to Finland are not included in the data, even if they enter cohabitation at that time. “Other” includes all other languages and combinations. This category is small because our sample consists only of individuals born in Finland

### Modelling

We apply discrete-time competing risk models for the hazard of entering a union with a partner of the type S-uniform, S-mixed, F-mixed, F-uniform and “other”, respectively, as a function of individuals’ ethnolinguistic identity and control variables. The cohabitation risks are estimated from age 18, in a discrete-time manner by calendar year. Individuals are right-censored at emigration, death, or at age 35, whichever comes first. The focus is on risk ratios between ego categories on having a partner of a specific ethnolinguistic affiliation. Because partner choice may exhibit gender differences, it is crucial to examine separately men and women. A couple model with covariates for male and female might come to mind, however such a strategy invokes complications from inter-partner dependence (cf. Elwert & Christakis, 2006). To avoid such issues that arise from counting couples twice, we instead estimate separate models for men and women and comment on differences effect size and magnitude.

### Control Variables

We include one control variable for ego’s education and a combination of highest educational level of ego’s parents, because higher education is associated with delayed entry into unions and generally different life course patterns (Jalovaara & Fasang, 2017; Jalovaara et al., 2019). For the egos, educational level is a time-varying variable categorized into primary, secondary and tertiary level of education. Parental education is a combination of mother’s and father’s highest observed level of education (primary, secondary or tertiary), resulting in nine categories.

We also include two contextual control variables at the local municipality (*kunta* or *kommun*) level. One is the proportion of the adult population aged 18–45 years in a municipality, for any given year, ever Swedish-registered. This accounts for the probability of meeting Swedish-registered partners, considering that 95%of all Swedish speakers reside along the west coast, and in the south, including the Helsinki metropolitan area, and they are much less mobile than Finnish speakers. Their number is higher in the South, while their share of the local population is larger along the west coast, and particularly in Ostrobothnia. As a consequence, mixed unions between Swedish and Finnish speakers are much more common in the southern part, and particularly in the Helsinki metropolitan area, than in the rest of the Swedish-speaking settlement area along the coast. The other contextual variable captures the yearly adult sex ratio at the municipality level, which previously has been linked to union formation (Schacht & Smith, 2017; Uggla & Mace, 2017). It is based on the proportion of men to women in the adult population aged 18–45 years. The age range was chosen to reflect that individuals aged up to 45 may still be considered part of the partner market for our 35 year olds. Both these contextual variables are lagged, so that it is where ego lived in the previous calendar year that may predict entry into cohabitation with a particular partner. The contextual variables are categorized into quintiles for easier interpretation. See Appendix for distributions and further details.

## Results

### Descriptive Statistics

**Fig. 1:**
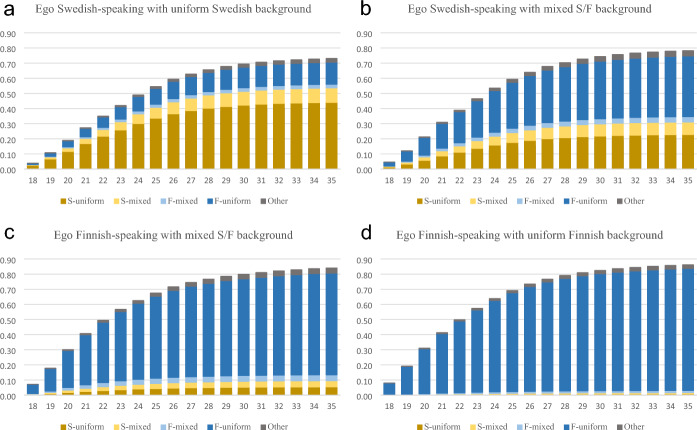
**a**–**d** Cumulative proportion of women’s first cohabiting partner’s ethnolinguistic background, by ego’s ethnolinguistic background (ego, mother and father). S/F-Mixed background refers to those with one parent who is a Swedish speaker and one who is a Finnish speaker, S/F-uniform refers where both parents are registered with the same language as ego. Other: partners with another mother tongue than Swedish or Finnish

**Fig. 2:**
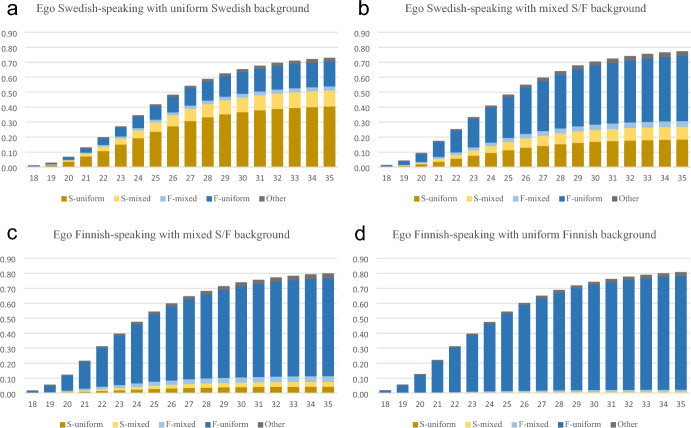
**a**–**d** Cumulative proportion of men’s first cohabiting partner’s ethnolinguistic background, by ego’s ethnolinguistic background (ego, mother and father). S/F-Mixed background refers to those with one parent who is a Swedish speaker and one who is a Finnish speaker, S/F-uniform refers where both parents are registered with the same language as ego. Other: partners with another mother tongue than Swedish or Finnish

We first construct cumulative proportions of ethnolinguistic affiliation of the first partner for each type of index person (S-uniform, S-mixed, F-mixed and, F-uniform) among women (Fig. 1a–d) and men (Fig. 2a–d). Note that the denominator of these cumulative proportions are based on all individuals at age 18, regardless of whether they are subsequently right-censored due to emigration or death.

Figure 1 shows that among Swedish-registered women with two Swedish-registered parents (i.e. S-uniform), about 44% had a first cohabiting partner with the same composition by age 35. For F-uniform women, the equivalent figure for having had a S-uniform male partner was only 0.7%. Among Finnish-registered women with two Finnish-registered parents (F-uniform), 81% had a F-uniform man as their first partner before 35. Swedish-registered women with mixed backgrounds (S-mixed) had lower rates of partnering with S-uniform men than S-uniform women, but were more likely to do so than their Finnish-registered counterparts. The partner choice of men shows a similar pattern to that of women, but Swedish-registered men are somewhat more likely to partner with a Finnish-registered person. Among Swedish-registered men with uniform Swedish background, about 41% partner with a similar (S-uniform) woman in their first cohabiting union. This figure is about 19% for Swedish-mixed men, and considerably lower among Finnish-registered men with mixed background (Fig. 2 and Table 2).

**Table 2:** Men’s first cohabiting partner by own ethnolinguistic background (*n* = 426 148)

		Partner’s ethnolinguistic background/censoring outcome								
Ego’s ethnolinguistic background		S-uniform (%)	S-mixed (%)	F-mixed (%)	F-uniform (%)	Other (%)	Never cohab. (%)	Emigrated (%)	Died (%)	Total
	S-uniform	42.0	11.0	2.9	17.1	2.1	13.3	10.6	0.9	15 878
	S-mixed	19.1	8.9	3.9	44.5	2.9	13.1	6.7	0.9	7 752
	F-mixed	4.6	3.3	3.9	67.0	2.7	15.0	2.4	1.1	8 116
	F-uniform	0.5	0.7	1.3	77.6	2.1	15.0	1.6	1.2	394 402

S/F-Mixed background refer to those with one parent who is Swedish-speaker and one who is a Finnish-speaker, S/F-uniform refer where both parents are registered with the same language as ego. If no cohabitation has occurred by age 35, the individual is recorded as never cohabitated. Emigrated and died are recorded as such if this event occurs before any cohabitation. Individuals who return to Finland are not included in the data, even if they enter cohabitation at that time. “Other” includes all other languages and combinations. This category is small because our sample consists only of individuals born in Finland

Tables 1 and 2 give the number of individuals who enter into each respective type of first cohabiting union, but also the number of individuals who were right-censored due to emigration, death, or never having cohabited by age 35. Swedish-registered women with uniform background are the most likely of all groups to emigrate before any other of these outcomes (17%), compared to only 3% of the Finnish-uniform women. The equivalent figures are approximately 11% for S-uniform men and 2% for F-uniform men. Some people remain in the country but have not had any cohabiting partner by the age of 35. Approximately 7% of S-uniform women, 8% of F-uniform women, 13% of S-uniform men and 15% of F-uniform men had not had any cohabiting partner by age 35.

### Competing Risks Models

Results of the competing risk regressions are summarized in Table 3 for women and Table 4 for men. Each column represents a different partner “risk” outcome; the independent variable, egos’ ethnolinguistic affiliation, is found in the rows. A Swedish-registered person with uniform Swedish background (S-uniform) is the ego reference category. We display results of unadjusted models and fully adjusted models side by side. Notably, results in adjusted models do not generally differ markedly from those in unadjusted models. We therefore focus on the adjusted models, but highlight examples where results diverge. Estimates for the control variables in the adjusted models are found in the Appendix.

**Table 3:** Competing risk showing subdistribution hazard rates (HR) for risks of first cohabiting partner’s ethnolinguistic background, women

		Partner’s ethnolinguistic background									
		S-uniform		S-mixed		F-mixed		F-uniform		Other	
		Unadj.	Adj.	Unadj.	Adj.	Unadj.	Adj.	Unadj.	Adj.	Unadj.	Adj.
Ego’s ethnolinguistic background	S-uniform	1	1	1	1	1	1	1	1	1	1
	S-mixed	0.42*	0.53*	0.82*	0.90*	1.41*	1.56*	3.17**	3.14*	1.36*	1.33*
	F-mixed	0.09*	0.14*	0.38*	0.52*	1.52*	1.97*	6.76*	6.32*	1.24*	1.44*
	F-uniform	0.01*	0.04*	0.08*	0.24*	0.54*	1.40*	9.28*	7.78*	0.90*	1.59*
	events	11 673		5 818		6 482		321 118		10 621	

Right-censored at emigration, death or at age 35. *denotes significance at the < 0.05 level. *Unadj*. unadjusted. *Adj*. denotes adjusted and controls for ego’s education (primary, secondary, tertiary, time-varying), parental education (mother and father, primary, secondary, tertiary, time-constant), proportion of ever Swedish-registered ages 18–45 in quintiles (time-varying, year -1. municipality-level), adult sex ratio ages 18–45 in quintiles (time-varying, year -1. municipality-level). Ethnolinguistic background is based on ego, their mother and father, e.g. S-mixed: Swedish-registered ego, with one parent who is Swedish-registered and one Finnish-registered

**Table 4:** Competing risk showing subdistribution hazard rates (HR) for risks of first cohabiting partner’s ethnolinguistic background, men

		Partner’s ethnolinguistic background									
		S-uniform		S-mixed		F-mixed		F-uniform		Other	
		Unadj.	Adj.	Unadj.	Adj.	Unadj.	Adj.	Unadj.	Adj.	Unadj.	Adj.
Ego’s ethnolinguistic background	S-uniform	1	1	1	1	1	1	1	1	1	1
	S-mixed	0.38*	0.45*	0.78*	0.82*	1.33*	1.41*	3.08*	2.91*	1.33*	1.36*
	F-mixed	0.08*	0.12*	0.27*	0.35*	1.30*	1.55*	5.61*	5.07*	1.22*	1.45*
	F-uniform	0.01*	0.03*	0.06*	0.16*	0.44*	1.06	7.19*	6.29*	0.93	1.55*
	events	10 426		5 508		6 306		317 487		9 137	

Right-censored at emigration, death or at age 35. *denotes significance at the < 0.05 level. *Unadj*. unadjusted. *Adj*. denotes adjusted and controls for ego’s education (primary, secondary, tertiary, time-varying), parental education (mother and father, primary, secondary, tertiary, time-constant), proportion of ever Swedish-registered ages 18–45 in quintiles (time-varying, year -1. municipality-level), adult sex ratio ages 18–45 in quintiles (time-varying, year -1. municipality-level). Ethnolinguistic background is based on ego, their mother and father, e.g. S-mixed: Swedish-registered ego, with one parent who is Swedish-registered and one Finnish-registered

As predicted, S-uniform women are the most likely to partner with a Swedish-registered man with uniform background, followed by S-mixed, F-mixed, and least likely are F-uniform women (Table 3). There is a quite notable difference in the magnitude of the hazard ratio (HR); Swedish-registered egos with mixed background, have 47% lower hazards (HR 0.53) to partner with an S-uniform man. There is an additional gap to Finnish-registered index persons with mixed backgrounds (HR 0.14), and even further to Finnish-registered with uniform background (HR 0.04). In other words, the higher degree of “Swedishness” ego exhibits, the more likely they are to partner with a Swedish-registered man with a uniform Swedish-speaking background. The partner choice of men showed the same pattern (Table 4).

We then consider who pairs with mixed background individuals. To partner with an S-mixed man is most likely among S-uniform and then S-mixed women. For these models, the difference in hazard ratios between S-uniform and S-mixed individuals are not as large as in the previous S-uniform partner model. However, there is still a divide between the groups identified above, including between Swedish-registered and Finnish-registered individuals with mixed background, or approximately 0.90 and 0.52. This goes counter to our prediction that S-mixed would be most likely to match with other S-mixed persons.

Moving to partners who are Finnish-registered mixed backgrounds, the most likely partner is another F-mixed person (95% higher hazards than a Swedish-registered woman with uniform background). This is in line with the hypothesis that matching will be based on own affiliation rather than on parental similarity only. For men, the HRs between Swedish-registered and Finnish-registered individuals with mixed backgrounds are more similar (41–55% higher hazards). Lastly, we report on the first cohabiting partners of F-uniform women and men. Unsurprisingly, F-uniform women are the most likely to partner with an F-uniform man, and least likely to partner with an S-uniform man (Table 3), and vice versa by sex for men (Table 4). The categories in between are in line with our predictions; the “more Swedish” the more likely to partner with another person with some Swedish-speaking belonging. In order to understand whether S-mixed and F-mixed differ statistically, we also run models with S-mixed as the reference category. There was a statistically significant difference in all models, except for partner outcome “other”.

Overall, the estimates from these models match fairly well with our predictions. Clear differences between our four categories emerged, i.e. between individuals who were (a) Swedish-registered with two Swedish-registered parents (b) Swedish-registered with mixed background, (c) Finnish-registered with mixed backgrounds, and (d) Finnish-registered with two Finnish-registered parents. It was interesting to note that for mixed background partners, the results varied between Swedish-registered and Finnish-registered individuals. The predictions were not met for the S-mixed (S-uniform were the most likely partners, followed by S-mixed). But for F-mixed individuals, F-mixed was indeed the most likely partner followed by S-mixed and only thereafter F-uniform. In the case of the male models, there was no statistical difference between S-uniform and F-uniform in partnering with an F-mixed woman.

The magnitude of effect sizes for first partner choice are often substantive; the hazard that a Finnish-speaking female ego with fully Finnish background will partner with the same background, is 678% higher than that a Swedish-speaking ego with fully Swedish background would partner with a F-uniform person. Hazards are also large between mixed categories (e.g. HR 3.14 vs. HR 6.32 for S-mixed and F-mixed in the female model), and far greater than magnitudes of education, for example between egos/parents who have tertiary versus secondary education (see Appendix).

### The Timing of the First Cohabitation

Entry into cohabitation with *any* partner, that is, regardless of partner’s ethnolinguistic affiliation, is faster for Finnish-registered individuals than for Swedish-registered individuals (see Appendix). However, by age 35, approximately equally many had entered the first cohabitation. In order to see if the hazard rate ratios are affected by differential timing into the first cohabitation between the groups, we ran separate models for ages 18–22 and ages 23–35 years. This cut-off was chosen to provide roughly half of the cohabiting events in each group. Overall, these results reveal that the estimates are highly similar in both age categories, and as compared with the main results’ entire age range (see the Appendix). Notwithstanding loss of power due to smaller sample sizes, conclusions remain the same. This indicates that partner choice is largely unaffected by any differences between ethnolinguistic groups in timing of the first cohabitation.

## Discussion

We have examined who partners with whom in first cohabiting unions (whether individuals were married or not) by individuals’ own and parental ethnolinguistic affiliation. To our knowledge, this is the first study that uses full population data across two generations to map minority-majority unions among two distinct ethnic groups that are ancestral to the country of study. Our contribution is important for understanding the prevalence of endogamy and exogamy in other contexts where minority groups are diminishing, indigenous languages risk extinction, or where some ethnic groups face numerical obstacles in search for a partner who shares their ethnicity or language. Specifying four combinations of ethnolinguistic background that encompass two endogamous and two mixed background combinations, and comparing partner choice across these combinations as we have done here, is a novel approach to unpack the dynamics by which social boundaries are maintained. In doing so, we also contribute with an analysis of distance between social groups in cohabitations (which may or may not be marriages). This approach reflects more diverse partner choice patterns than focusing on more stable (marital) unions only.

A key insight from the results is the importance of considering group belonging across two generations. In most of the models, we saw a pattern of differences in magnitude between Swedish-registered persons with uniform Swedish background, Swedish-registered with mixed background, Finnish-registered with mixed background, and Finnish-registered with uniform Finnish background. For instance, as compared with Swedish-registered persons with uniform Swedish background, those with mixed background are considerably less likely to partner with a Swedish-registered person with uniform Swedish background. This pattern was found among both men and women. In order words, while ego’s affiliation proved important, not all Swedish-registered individuals are equal in their first partner choice risk. The parental ethnolinguistic affiliations are consequently needed to understand the full complexity of partner choices in this minority-majority context.

These patterns are consistent with the matching hypothesis, i.e. that partners choose others who are like themselves on specific traits. Yet, it is hard to discern whether the partner choice patterns result because individuals share communication (language), similar values or because of other factors that could promote unions between similar individuals. An alternative, but not mutually exclusive, explanation for these assortative mating patterns is that opportunities to find others like oneself play a major role. For instance, individuals with mixed backgrounds are more likely to reside in mixed areas, such as the Helsinki region, and may therefore be more likely to meet other mixed background people who live there. However, controlling for the share of Swedish speakers at the local residential level did not affect our results markedly. In most adjusted models, the differences between ego types were attenuated but the order was the same. The exception were the models for Finnish-registered with mixed backgrounds, which fluctuated with controls for education, local language background and the proportion of men in the area.

The clear distinction in partner choice between Swedish speakers with uniform Swedish background and mixed background, suggests that a part of the Swedish speaking minority has an especially tight-knit community. Among Swedish speakers with uniform background, endogamous partnership is more likely to be transmitted. Once such endogamous unions are formed, they are less likely to break down and lead to other (potentially exogamous) partnerships (Saarela & Finnäs, 2014). It has been proposed that low geographical mobility, and a high degree of social integration is a contributing factor for the considerably lower divorce rates among endogamous Swedish-speaking couples as compared with Finnish-speaking couples (Finnäs, 1997). Interestingly, we noted that Swedish registered individuals did not have higher raw rates of being “never partnered” by age 35. In fact, Finnish speaking men with endogamous backgrounds, were the least likely to have had a cohabiting union. This goes against the idea that being the minority makes it less likely to find a partner, at least for the coresidential unions we examine here.

Our data indicated that individuals with mixed background clustered in between endogamous pairs of either ethnolinguistic group. There were some differences between the Swedish-registered and Finnish-registered individuals in who the most likely partner of a mixed background person was; for F-mixed, the most likely partner was another F-mixed, rather than Finnish-registered person with uniform Finnish background. This implies that having mixed background as a Finnish-registered person is a distinct sub-group and that it is somewhat maintained in the next generation, at least with respect to first unions. In the international literature, individuals with mixed ethnic background are often considered to have a more blurred identity than those from endogamous majority or minority unions, and may identify more with national than ethnic identity (Lewin-Epstein & Cohen, 2019; Song, 2010). A parallel between the Finnish case can be drawn to individuals born to one Jewish and one non-Jewish parent in the USA, among whom religious exogamous marriage is much more common than children from endogamous Jewish marriages (Fishman, 2004). Children of mixed marriages are also much less likely to identify as Jewish. Sociologists have recognized that ethnic categorizations are not static, but continually reformulated (Lieberson & Waters, 1986). This may especially be the case for individuals who live and interact closely with another ethnic group, such as in the event of intermarriage (Petts & Petts, 2019). In Finland, language (and bilingualism) adds a practical aspect to such continual reformulation. Yet, when parents choose a Swedish ethnolinguistic affiliation for their children, this often entails attending a Swedish speaking school and being part of the Swedish speaking community. How much of a person’s identity that comes from having one Finnish speaking parent who did not grow up as part of that community, is difficult to ascertain without qualitative data. However, our partner choice analysis provides an indication of closeness between mixed background individuals and persons in the “other” group.

It should be highlighted that a noticeable proportion of Swedish-registered individuals emigrated before forming a cohabiting union in Finland (approximately 17% and 11% of women and men, respectively). The most common destination is the neighbouring country Sweden. During the past 20 years, two thirds of all emigration of Swedish-speaking Finns have been in the direction of Sweden, and the net emigration loss during the same period amounts to approximately 3,700 Swedish-registered persons (Saarela, 2021). These migration patterns have potential implications on our results. The non-movers we are capturing are either Swedish speakers who are particularly well-integrated in the Swedish-speaking community, or conversely, Swedish speakers who are more open to interacting with the Finnish-speaking society and, thus, possibly also more open to a Finnish-speaking partner. In order to reliably capture the complete cohabitation data we had to focus on individuals in the stationary population, but how migration can be a cause of a limited partner market can be explored by future studies.

We have examined *first* partner choice (whether a marital union or not) but in a context of serial monogamy, some individuals will move on from the partnership observed here. From another Finnish study with roughly the similar time frame, 42% of the cohabitations had proceeded to marriage without children, 23% to cohabitation with children, and the remaining 35% had ended in separation eight years after union entry (Saarela & Finnäs, 2014). The proportion of cohabiting unions that become childbearing unions of course impact ethnolinguistic inheritance and composition of the next generation. Future studies could investigate the partner choice in childbearing unions, and in particular how it varies by the person’s previous partner choice (cf. Obućina, 2016), in order to understand whether there are patterned differences by union order. Also, while the role of partner’s education has been beyond the scope of this article, future studies could consider ethnicity and education in unions to gain even more detailed insights of partner choice across different domains. In our study context it is difficult to draw a clear line between non-marital and marital unions, primarily because many of the former are eventually turned into the latter. However, we still know little about what unobserved individual level characteristics of minority and majority group partners may play a role for these results. If data on personality were available, it could be interesting to see whether psychological aspects of wanting to adhere to social (in-group) norms play a part in the partner choice. Future studies could fruitfully also explore more fine-grained socioeconomic characteristics, such as employment type, income and other factors that may be related to whether a person moves to a different area to seek employment, or start studies, which indirectly may affect partner choice. While it is beyond the scope of this article, a more detailed examination of an individual’s life course and internal migration, may thus help to shed light on how local areas influence the choice of partner.

When seeking to generalize these findings a few factors are important to bear in mind. While some Swedish-speaking regions display strong social integration, one can equally characterize Finland as a context where social boundaries are weak and ethnically based discrimination is not prevalent. Finland is also a very homogenous country because immigration was almost non-existent until a few decades ago. Rather than diverse immigrant origins, the main heterogeneity consists of the two ancestral groups examined here. These features stand in clear contrast to the diversity in other European countries that stems from long-term immigration, and the stigma and discrimination that is often associated with ethnic or racial intermarriages in, e.g. the USA. Despite such differences, we believe that our findings can be informative for understanding social relationships between majority-minority groups. The low social boundaries in Finland remove unnecessary constraints and allow young people to form unions with those they naturally come in contact with, regardless of social background.

Another crucial point is that there are both similarities and differences between ethnic and language groups (Stevens & Schoen, 1988). Finnish speakers and Swedish speakers are two distinct ethnicities, but, bilingualism aside, they are divided by the practicalities of language. It is likely that the relatively small differences between any combinations of Swedish-registered persons in having a Swedish-registered partner, reflects the importance of sharing a common language. Having some connection to a minority group may lead individuals to identify with this group if it is seen as desirable, and if it carries some material, apart from only symbolic, benefit (Lieberson, 1985). It has been argued that Swedish-registration is one such entity, which may be why Swedish-registration of children is more common than Finnish-registration in mixed unions (Finnäs & O’Leary, 2003). Regardless of the explanation, we conclude that the binary registration system in Finland, where parents have to choose *one* registered language for their children, appears to reflect the social group of their child at young adult age, and that it is a good predictor of subsequent partner choice.

A relatively large group of Swedish speakers with endogamous background might keep partnering endogamously and maintain Swedish identity, but Swedish speakers with exogamous background will have a large influence over the relative composition of ethnolinguistic groups in Finland in the future. It has not been our objective to disentangle the mechanisms behind partner choice patterns, but where the data exist, our detailed taxonomy could fruitfully be applied to other minority-majority contexts, to better understand how social boundaries evolve. Others have argued that with the present patterns concerning ethnic partner choice and language registration of children, the Swedish speaking minority, is not under immediate risk of extinction (Saarela et al., 2020). Yet, future research might also examine more closely the role of gender in passing on ethnolinguistic identity, whether it is the same in the minority and majority group, and the relative role of parental versus ego’s own affiliation across contexts.

## Figures and Tables

**Fig. 3:**
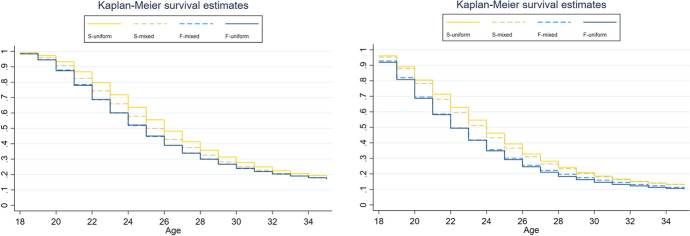
**a**, **b** Kaplan-Meier plots for any partner (first cohabitation), by ego’s ethnolinguistic background, for any partner before age 35, women (left) and men (right)

## Appendix

See Fig. 3, Tables 5, 6, 7, 8, 9, 10 and 11.

**Table 5:** Competing risk showing subdistribution hazard rates (HR) for risks of first cohabiting partner’s ethnolinguistic background, women

Women		S-uniform	S-mixed	F-mixed	F-uniform	Other
Ego’s ethnolinguistic background	S-uniform	1	1	1	1	1
	S-mixed	0.53*	0.91*	1.56*	3.14*	1.33*
	F-mixed	0.14*	0.52*	1.96*	6.32*	1.44*
	F-uniform	0.04*	0.24*	1.40*	7.78*	1.58*
Ego education (Secondary)	Primary	0.64*	0.59*	0.73*	0.86*	0.76*
	Tertiary	2.76*	2.56*	2.49*	1.71*	2.27*
Parental education (Both secondary)	Primary-Primary	0.97	1.15*	1.10*	0.99*	0.84*
	Primary-Secondary	0.97	1.13*	1.07	1.00	0.93
	Primary-Tertiary	0.80*	0.90	0.89	0.81*	1.06
	Secondary-Primary	0.96	1.07	1.07	0.99	0.90*
	Secondary-Tertiary	0.78*	0.99	0.96	0.80*	1.16*
	Tertiary-Primary	0.84*	1.04	0.93	0.84*	1.10
	Tertiary-Secondary	0.80*	1.08	0.95	0.85*	1.17*
	Tertiary-Tertiary	0.70*	1.09	0.82*	0.69*	1.26*
Proportion Swedish-registered, municipality year-1 (3rd quintile)	1st quintile	0.07*	0.10*	0.37*	0.93*	0.65*
	2nd quintile	0.50*	0.45*	0.54*	1.00	0.82*
	4th quintile	3.31*	2.31*	2.24*	0.97*	1.28*
	5th quintile	56.19*	15.43*	7.88*	0.77*	2.60*
Proportion male, municipality year-1 (3rd quintile)	1st quintile	0.72*	1.30*	1.26*	1.40*	2.13*
	2nd quintile	1.02	1.23*	1.41*	1.18*	1.45*
	4th quintile	0.96	1.09	0.86	0.93*	0.78*
	5th quintile	1.02	0.88	0.80	0.86*	0.80

Parental education is in the order of mother:father. Ethnolinguistic background is based on ego, their mother and father, e.g. S-mixed: Swedish-registered ego, with one parent who is Swedish-registered and one Finnish-registered. S/F-uniform: both parents are registered as either S or F.

**Table 6:** Competing risk showing subdistribution hazard rates (HR) for risks of first cohabiting partner’s ethnolinguistic background, men

Men		S-uniform	S-mixed	F-mixed	F-uniform	Other
Ego’s ethnolinguistic background	S-uniform	1	1	1	1	1
	S-mixed	0.45*	0.82*	1.41*	2.91*	1.36*
	F-mixed	0.12*	0.35*	1.55*	5.07*	1.45*
	F-uniform	0.03*	0.16*	1.06	6.29*	1.55*
Ego education (Secondary)	Primary	0.84*	0.85*	1.07*	0.95*	0.97
	Tertiary	1.98*	2.12*	1.97*	1.48*	2.36*
Parental education (Both secondary)	Primary-Primary	0.94	1.04	1.07	0.97*	0.86*
	Primary-Secondary	0.96	1.01	1.00	0.97*	0.93
	Primary-Tertiary	0.74*	0.96	0.92	0.86*	1.04
	Secondary-Primary	0.95	1.01	1.01	1.00	0.91*
	Secondary-Tertiary	0.84*	0.84*	0.86*	0.86*	1.21*
	Tertiary-Primary	0.91*	0.96	1.00	0.92*	1.09
	Tertiary-Secondary	0.84*	0.96	0.85*	0.93*	1.16*
	Tertiary-Tertiary	0.69*	1.01	0.81*	0.80*	1.31*
Proportion Swedish-registered, municipality year-1 (3rd quintile)	1st quintile	0.13*	0.17*	0.57*	0.90*	0.61*
	2nd quintile	0.44*	0.60*	0.68*	1.00	0.81*
	4th quintile	2.52*	2.56*	1.88*	0.93*	1.35*
	5th quintile	32.01*	13.87*	6.70*	0.79*	2.16*
Proportion male, municipality year-1 (3rd quintile)	1st quintile	0.85*	1.27*	1.10	1.70*	1.30*
	2nd quintile	1.14*	1.25*	1.13	1.23*	1.45*
	4th quintile	0.99	0.98	0.74*	0.87*	1.16*
	5th quintile	1.00	0.85	0.48*	0.70*	0.81

Parental education is in the order of mother:father. Ethnolinguistic background is based on ego, their mother and father, e.g. S-mixed: Swedish-registered ego, with one parent who is Swedish-registered and one Finnish-registered. S/F-uniform: both parents are registered as either S or F.

**Table 7:** Competing risk showing subdistribution hazard rates (HR) for risks of first cohabiting partner’s ethnolinguistic background, women

		Risk of first cohabiting partner’s ethnolinguistic background									
		S-uniform		S-mixed		F-mixed		F-uniform		Other	
		Younger	Older	Younger	Older	Younger	Older	Younger	Older	Younger	Older
Ego’s ethnolinguistic background	S-uniform	1	1	1	1	1	1	1	1	1	1
	S-mixed	0.55*	0.55*	0.96	0.85*	1.54*	1.55*	3.08*	2.88*	1.44*	1.27*
	F-mixed	0.16*	0.14*	0.64*	0.41*	2.12*	1.70*	5.96*	5.42*	1.74*	1.35*
	F-uniform	0.05*	0.04*	0.27*	0.20*	1.38*	1.40*	6.97*	6.43*	1.91*	1.42*
	Events	Younger: 5652 Older: 6021		Younger: 2985 Older: 2833		Younger: 3735 Older: 2747		Younger: 188148 Older: 132,970		Younger: 3818 Older: 6803	

Age-stratified. Younger: 18–22 years, Older: 23–35 years. Right-censored at emigration, death or at age 23/35. *denotes significant at the <0.05 level. Controls for ego’s education (primary, secondary, tertiary, time-varying), parental education (mother and father, primary, secondary, tertiary, time-constant), proportion of ever Swedish-registered ages 18–45 in quintiles (time-varying, year -1. municipality-level), adult sex ratio ages 18–45 in quintiles (time-varying, year -1. municipality-level). Ethnolinguistic background is based on ego, their mother and father, e.g. S-mixed: Swedish-registered ego, with one parent Swedish-registered and one Finnish-registered

**Table 8:** Competing risk showing subdistribution hazard rates (HR) for risks of first cohabiting partner’s ethnolinguistic background, men

		Risk of first cohabiting partner’s ethnolinguistic background									
		S-uniform		S-mixed		F-mixed		F-uniform		Other	
		Younger	Older	Younger	Older	Younger	Older	Younger	Older	Younger	Older
Ego’s ethnolinguistic background	S-uniform	1	1	1	1	1	1	1	1	1	1
	S-mixed	0.51*	0.46*	0.83*	0.84*	1.54*	1.37*	3.11*	2.72*	1.61*	1.34*
	F-mixed	0.14*	0.12*	0.41*	0.33*	1.67’	1.54*	5.29*	4.50*	1.95*	1.39*
	F-uniform	0.04*	0.03*	0.19*	0.16*	1.12	1.08	6.29*	5.48*	1.78*	1.54*
	Events	Younger: 3012 Older: 7414		Younger:1802 Older: 3706		Younger: 2434 Older:3873		Younger: 123664 Older: 193823		Younger: 1656 Older: 7481	

Age-stratified. Younger: 18–22 years, Older: 23–35 years. Right-censored at emigration, death or at age 23/35. *denotes significant at the <0.05 level. Controls for ego’s education (primary, secondary, tertiary, time-varying), parental education (mother and father, primary, secondary, tertiary, time-constant), proportion of ever Swedish-registered ages 18-45 in quintiles (time-varying, year -1. municipality-level), adult sex ratio ages 18-45 in quintiles (time-varying, year -1. municipality-level). Ethnolinguistic background is based on ego, their mother and father, e.g. S-mixed: Swedish-registered ego, with one parent Swedish-registered and one Finnish-registered

**Table 9:** Egos highest level of education (time-varying) and ego’s ethnolinguistic affiliation and background

		Ego’s highest education		
		Primary	Seconday	Tertiary
Ego’s ethnolinguistic background	S-uniform	22.2%	60.7%	17.1%
	S-mixed	25.5%	58.8%	15.2%
	F-mixed	30.5%	57.5%	12.0%
	F-uniform	26.6%	59.6%	13.9%

**Table 10:** Egos highest level of education of ego’s mother and ego’s father, in that order

		Parental level of education (mother’s:father’s)								
		PriPri	PriSec	PriTer	SecPri	SecSec	SecTer	TerPri	TerSec	TerTer
Ego’s ethnolinguistic background	S-uniform	16.2%	8.0%	4.1%	13.3%	14.1%	9.8%	4.8%	7.4%	22.1%
	S-mixed	12.1%	8.0%	5.6%	10.4%	11.5%	10.4%	5.4%	8.5%	28.2%
	F-mixed	16.5%	10.6%	5.4%	12.8%	14.3%	9.2%	5.0%	7.2%	19.1%
	F-uniform	14.4%	10.4%	3.4%	14.1%	19.4%	8.7%	4.2%	7.8%	17.5%

Pri denotes Primary, Sec-seconday, Ter-tertiary education, by ego’s ethnolinguistic affiliation and background (order ego:mother:father)

**Table 11:** Proportion ever Swedish-registered 18–45 years, proportion male 18–45 years at the municipality level, in quintiles, time varying, by ego’s ethnolinguistic affiliation and background (order ego:mother:father).

		Proportion Swedish					Proportion male				
		1st	2nd	3rd	4th	5th	1st	2nd	3rd	4th	5th
	S-uniform	0.0%	0.0%	0.2%	0.5%	99.3%	59.0%	17.7%	14.7%	6.9%	1.7%
	S-mixed	0.0%	0.5%	1.5%	3.6%	94.5%	77.2%	10.5%	7.7%	3.8%	0.8%
	F-mixed	0.7%	2.5%	5.7%	9.6%	81.5%	80.6%	10.2%	5.9%	2.5%	0.7%
	F-uniform	8.3%	17.6%	23.1%	19.5%	31.5%	62.1%	15.4%	10.7	7.4%	4.5%
